# Supplementary figures and images for: Herpes simplex virus co-infection facilitates rolling circle replication of the adeno-associated virus genome
Source: PLoS Pathog. 2021 Jun 1;17(6):e1009638. doi: 10.1371/journal.ppat.1009638 (PMC8195378; doi:10.1371/journal.ppat.1009638)

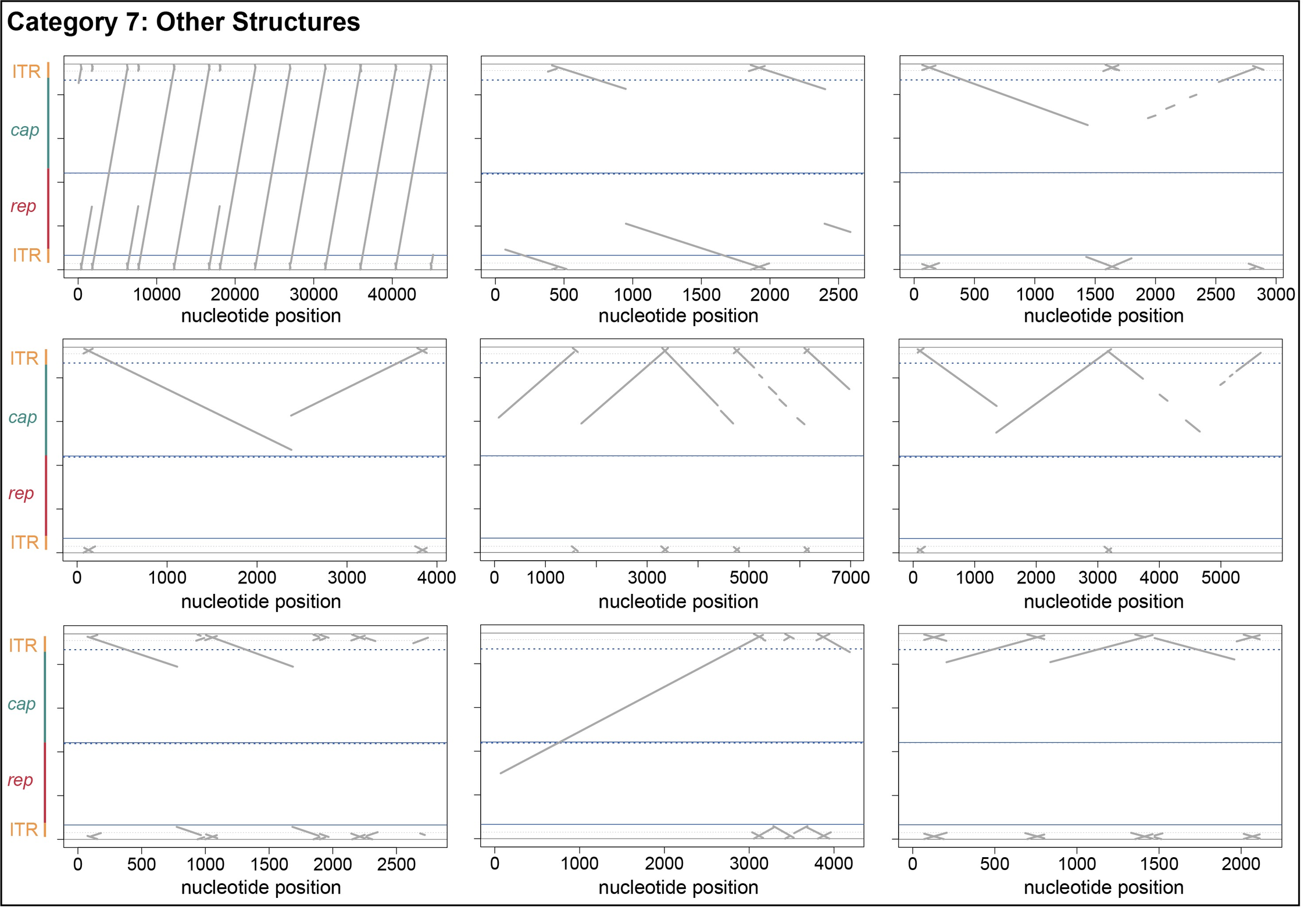

Supplement: S1 Fig — Dot plots of individual reads assigned to category 7 (other structures) from AAV2/HSV-1 co-infected BJ cells are shown. Extrachromosomal DNA was isolated at 12 hpi from BJ cells infected with AAV2 (gcp/ cell = 20’000) and HSV-1 (pfu/cell = 1). (TIF) [file ppat.1009638.s001.tif]

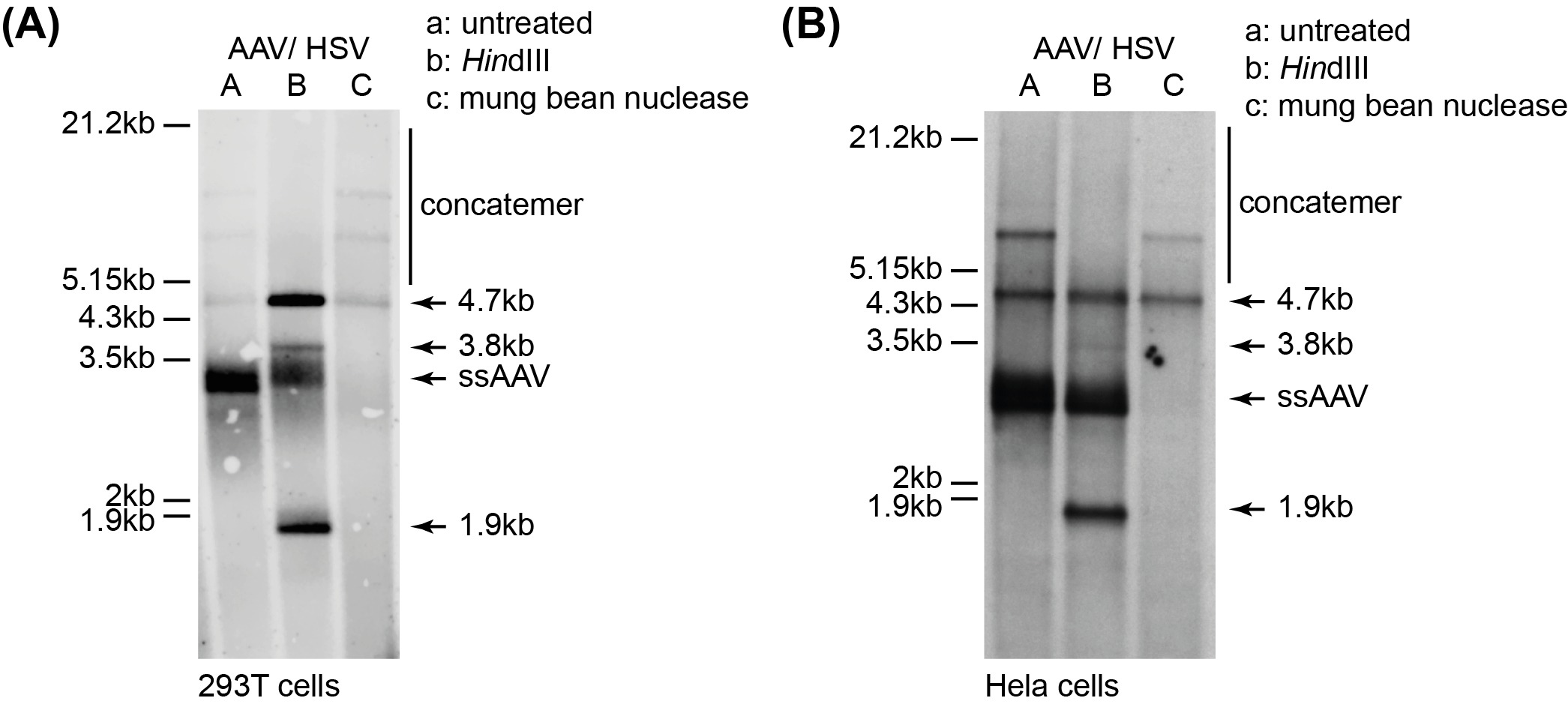

Supplement: S2 Fig — Southern blot of Hirt DNA extracted at 15 hpi from 293T cells (A) or at 20 hpi from Hela cells (B) co-infected with AAV2 (gcp/ cell = 20’000) and HSV-1 (pfu/cell = 1) showing untreated (a), HindIII digested (b) or mung bean nuclease treated (c) samples. (TIF) [file ppat.1009638.s002.tif]

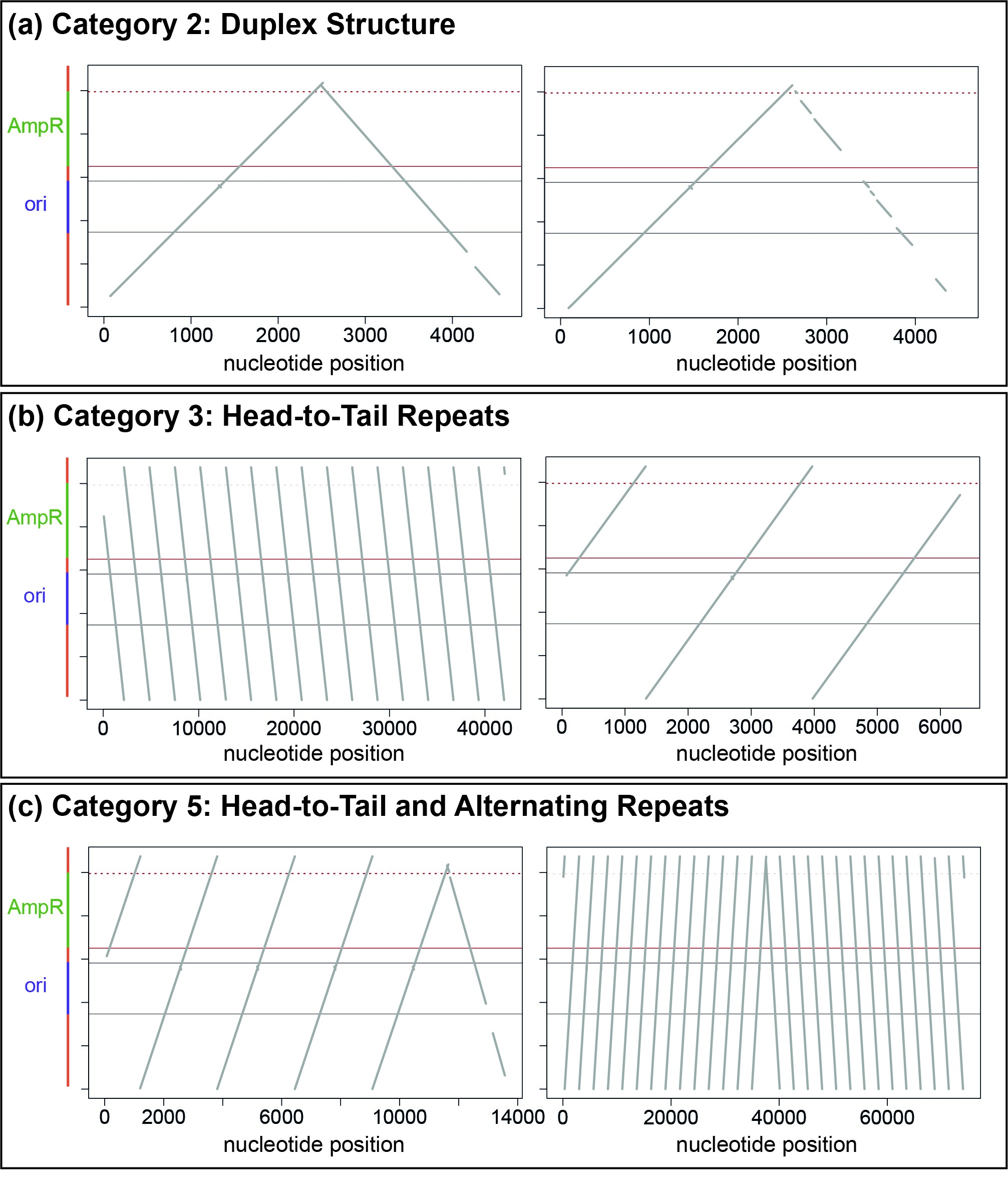

Supplement: S3 Fig — Dot plots of two individual reads per category of DNA isolated from Phi29 polymerase amplified pUC19. (TIF) [file ppat.1009638.s003.tif]
