# Supplementary material for: Herpes simplex virus co-infection facilitates rolling circle replication of the adeno-associated virus genome
Source: PLoS Pathog. 2021 Jun 1;17(6):e1009638. doi: 10.1371/journal.ppat.1009638 (PMC8195378; doi:10.1371/journal.ppat.1009638)
Supplement: S1 Table — (DOCX) [file ppat.1009638.s004.docx]

**Table S1.** Read analysis data from genomes isolated from AAV201 single- or HSV-1 co-infected BJ cells at 12 hpi.

| **Category:** | | **1** | **2** | **3** | **4** | **5** | **6** | **7** |  |
| --- | --- | --- | --- | --- | --- | --- | --- | --- | --- |
| **Sample** | | **Monomer** | **Duplex** | **Head-to-Tail Repeats** | **Alternating Repeats** | **Head-to-Tail and Alternating Repeats** | **ITR Repeats** | **Others** |  |
|  | **AAV gcp/ cell** | **ratio** | **ratio** | **ratio** | **ratio** | **ratio** | **ratio** | **ratio** | **total reads** |
| **AAV201** | **20k** | 0.632 | 0.098 | 0.000 | 0.000 | 0.000 | 0.127 | 0.142 | 204 |
| **AAV201/ HSV-1** | **20k** | 0.162 | 0.221 | 0.235 | 0.025 | 0.054 | 0.108 | 0.196 | 204 |
