# Supplementary material for: Herpes simplex virus co-infection facilitates rolling circle replication of the adeno-associated virus genome
Source: PLoS Pathog. 2021 Jun 1;17(6):e1009638. doi: 10.1371/journal.ppat.1009638 (PMC8195378; doi:10.1371/journal.ppat.1009638)
Supplement: S2 Table — The number of AAV genome copies per category was assessed. The same dataset to generate Table 1 was used. (DOCX) [file ppat.1009638.s005.docx]

**Table S2.** Read analysis data from genomes isolated from AAV2 single- or HSV-1 co-infected BJ cells at 12 hpi. The number of AAV genome copies per category was assessed. The same dataset to generate Table 1 was used

| **Category:** | | **1** | **2** | **3** | **4** | **5** |  |
| --- | --- | --- | --- | --- | --- | --- | --- |
| **Sample** | | **Monomer** | **Duplex** | **Head-to-Tail Repeats** | **Alternating Repeats** | **Head-to-Tail and Alternating Repeats** |  |
|  | **AAV gcp/ cell** | **ratio** | **ratio** | **ratio** | **ratio** | **ratio** | **total genome copy number** |
| **AAV2** | **20k** | 0.750 | 0.179 | 0.054 | 0.018 | 0.000 | 168 |
| **AAV2/ HSV-1** | **20k** | 0.196 | 0.170 | 0.330 | 0.085 | 0.219 | 342 |
| **AAV2** | **500** | 0.231 | 0.769 | 0.000 | 0.000 | 0.000 | 13 |
| **AAV2/ HSV-1** | **500** | 0.086 | 0.402 | 0.308 | 0.039 | 0.164 | 383 |
