# Supplementary material for: Herpes simplex virus co-infection facilitates rolling circle replication of the adeno-associated virus genome
Source: PLoS Pathog. 2021 Jun 1;17(6):e1009638. doi: 10.1371/journal.ppat.1009638 (PMC8195378; doi:10.1371/journal.ppat.1009638)
Supplement: S3 Table — “A” and “V” indicate the orientation as observed in the dotplots. For AAV-sequences “A” indicates the presence of a covalent link at the 5’-end (cap-side) whereas “V” would indicate the presence of a covalent link at the 3’-end (rep-side). (DOCX) [file ppat.1009638.s006.docx]

**Table S3.** Further read analysis data from extrachromosomal MVM sequences isolated from MVM infected A9 cells or AAV2 sequences from AAV2 single- or HSV-1 co-infected BJ cells of category 2. “A” and “V” indicate the orientation as observed in the dotplots. For AAV-sequences “A” indicates the presence of a covalent link at the 5’-end (cap-side) whereas “V” would indicate the presence of a covalent link at the 3’-end (rep-side).

| **Category:** | | | **2** | **2a** | **2b** |  |
| --- | --- | --- | --- | --- | --- | --- |
| **Sample** | | | **2 Duplex Structure (total)** | **2a Duplex Structure "A"** | **2b Duplex Structure "V"** |  |
| **Infection** | **MOI** | **harvest** | **ratio** | **ratio** | **ratio** | **total reads** |
| **MVM** | **1k** | **16hpi** | 0.730 | 0.050 | 0.680 | 200 |
| **MVM** | **1k** | **20hpi** | 0.795 | 0.065 | 0.730 | 200 |
| **MVM** | **10k** | **16hpi** | 0.767 | 0.087 | 0.680 | 103 |
| **MVM** | **10k** | **20hpi** | 0.670 | 0.100 | 0.570 | 200 |
| **AAV2** | **20k** | **12hpi** | 0.080 | 0.040 | 0.040 | 200 |
| **AAV2/ HSV-1** | **20k/ 1** | **12hpi** | 0.170 | 0.105 | 0.065 | 200 |
| **AAV2** | **500** | **12hpi** | 0.333 | 0.083 | 0.250 | 12 |
| **AAV2/ HSV-1** | **500/ 1** | **12hpi** | 0.385 | 0.095 | 0.290 | 200 |
