# Supplementary material for: Herpes simplex virus co-infection facilitates rolling circle replication of the adeno-associated virus genome
Source: PLoS Pathog. 2021 Jun 1;17(6):e1009638. doi: 10.1371/journal.ppat.1009638 (PMC8195378; doi:10.1371/journal.ppat.1009638)
Supplement: S1 Bioinformatic code — (DOCX) [file ppat.1009638.s007.docx]

Bioinformatic Code S1 Bioinformatic code used to analyze and plot the nanopore sequencing data.

#####################################################################################

### on UNIX platform on UNIX platform on UNIX platform on UNIX platform on UNIX platform on UNIX ###

#####################################################################################

Change nanopore reads from fastq- to fasta-format using seqkit:

[W Shen, S Le, Y Li, F Hu. "SeqKit: a cross-platform and ultrafast toolkit for FASTA/Q file manipulation." PLOS ONE. doi:10.1371/journal.pone.0163962.] download and install: https://bioinf.shenwei.me/seqkit/download/

# execute command:

seqkit fq2fa <path_to_fastq_files.fastq> -o <path_to_fasta_files.fasta>

Alignment of reads in fasta-format with blastn against ref_seq:

[Christiam Camacho 1 , George Coulouris, Vahram Avagyan, Ning Ma, Jason Papadopoulos, Kevin Bealer, Thomas L Madden "BLAST+: architecture and applications" BMC Bioinformatics doi: 10.1186/1471-2105-10-421.] download and install: https://ftp.ncbi.nlm.nih.gov/blast/executables/blast+/LATEST/

# execute command:

blastn -word_size 11 -reward 2 -penalty -3 -query <path_to_fastq_files.fasta> -db <path_to_blastn_db> -outfmt "7 qacc sacc evalue qstart qend sstart send qlen" -out <path_to_outfile>

# remove lines with non-hits and comments from blastn-output and save to text-file:

grep -v '#' <path_to_outfile> > <path_to_outfile.txt>

#####################################################################################

### in R in R in R in R in R in R in R in R in R in R in R in R in R in R in R in R in R in R in R in R in R ###

#####################################################################################

Read values of blastn hit table into R and store as dataframe ‘sample_name’:

sample_name <- read.table("<path_to_outfile.txt>", sep="\t", col.names=c("read_ID", "sub_name", "e_val", "que_beg", "que_end", "sub_beg", "sub_end", "que_length"))

Reduce dataframe for hits with e-value smaller than 0.1:

sample_name <- sample_name[sample_name$e_val < 0.1, ]

Order dataframe according to the read_ID:

sample_name <- sample_name[order(sample_name$read_ID), ]

Generate column in dataframe with rep_hits:

########################################################################################

# define function to generate vector w/ identical read nummber for all hits of same read:

# USAGE: df$rep_hits <- rep.hits(df)

rep.hits <- function(df) {

x <- c(); n <- tabulate(as.factor(df$read_ID));

for (i in 1:length(n)) {x <- append(x, rep(i, n[i]))}

return(x)

}

#=======================================================================================

Apply rep.hits-function on dataframe:

sample_name$rep_hits <- rep.hits(sample_name)

Generate column in dataframe with tot_hits:

#########################################################################################

# define function to generate vector w/ total hit length for each read:

# USAGE: df$tot_hits <- tot.hits(df)

tot.hits <- function (df) {

sum_read <- c(); sum_vect <- c();

for (i in 1:max(df$rep_hits)) {

sum_read <- sum(df[df$rep_hits == i,]$que_end - df[df$rep_hits == i,]$que_beg);

sum_vect <- append(sum_vect, rep(sum_read, tabulate(df$rep_hits)[i]));

}

return(sum_vect);

}

#========================================================================================

Apply tot.hits-function on dataframe:

sample_name$tot_hits <- tot.hits(sample_name)

Reduce dataframe for tot_hits larger than 3000nt:

sample_name <- sample_name[sample_name$tot_hits > 3000, ]

Generate NEW rep_hits column in dataframe:

sample_name$rep_hits <- rep.hits(sample_name)

Define functions to draw dotplots:

#########################################################################################

# define function to draw one dotplot of "wtAAV" blast hits from one nanopore read:

# USAGE: dot.wt(df)

dot.wt <- function (df) {

plot(NULL, xlim=c(0, df$que_length[1]), ylim=c(0, 4700), xlab="Query", ylab="Subject", main=df$read_ID[1]);

# draw annotation on dt plot:

abline(h=329, col="blue"); abline(h=2194, lty=2, col="blue"); abline(h=2211,col="blue"); abline(h=4337, lty=2, col="blue");

text(1,1261,"rep", cex=0.5, col="blue"); text(1,3274,"cap", cex=0.5,col="blue")

abline(h=0, lwd=0.5); abline(h=150, lty=2, lwd=0.5); abline(h=4550, lty=2, lwd=0.5); abline(h=4700, lwd=0.5);

text(1,50,"ITR", cex=0.5); text(1,4600,"ITR", cex=0.5)

# draw individual hits:

for (i in 1:length(df$read_ID)) {

segments(df$que_beg[i], df$sub_beg[i], df$que_end[i], df$sub_end[i], col="darkgrey", lwd=3);

}

}

#========================================================================================

#########################################################################################

# define function to draw dotplots blast hits on all nanopore reads from one sample:

# USAGE: dot.all(df, dot.wt)

dot.all <- function (df, func) {

for (j in 1:max(df$rep_hits)) {

temp_df <- df[df$rep_hits == j,];

func(temp_df);

}

}

#========================================================================================

Generation of dotplots from all nanopore reads redirected into a pdf-file:

pdf("<path_to_output.pdf>", paper="a4", width=7.3, height=10.7)

par(mfrow=c(4,2))

dot.all(sample_name, dot.wt)

dev.off()
